# First record of the soldier fly genus *Beris* Latreille (Diptera, Stratiomyidae) from Korea, with designation of two new synonyms

**DOI:** 10.3897/BDJ.10.e80487

**Published:** 2022-08-01

**Authors:** Junho Lee, Sang Jae Suh

**Affiliations:** 1 School of Applied Biosciences, Kyungpook National University, Daegu, Republic of Korea School of Applied Biosciences, Kyungpook National University Daegu Republic of Korea; 2 Institute of Plant Medicine, Kyungpook National University, Daegu, Republic of Korea Institute of Plant Medicine, Kyungpook National University Daegu Republic of Korea; 3 Department of Plant Protection and Quarantine, Kyungpook National University, Daegu, Republic of Korea Department of Plant Protection and Quarantine, Kyungpook National University Daegu Republic of Korea

**Keywords:** Beridinae, *Beris*, Korea, new synonyms, Stratiomyidae, taxonomy

## Abstract

**Background:**

The soldier fly subfamily Beridinae in the family Stratiomyidae contains approximately 280 known species, these being distributed across nearly all biogeographical regions with the exception of the polar regions. On the Korean Peninsula, the species diversity of this subfamily has been poorly examined to date, as is reflected in the National species list of Korea by the National Institute of Biological Resources.

**New information:**

In this paper, the soldier fly genus *Beris* Latreille is reported for the first time from Korea, based on observations of the following three species: *B.fuscipes* Meigen, 1820, *B.hildebrandtae* Pleske, 1930 and *B.hirotui* Ôuchi, 1943. Additionally, the authors propose two nomenclatural acts: 1) *B.angustifacies* Nagatomi and Tanaka, 1972 **syn. nov.** = *B.hildebrandtae* Pleske, 1930; 2) *B.liaoningana* Cui, Li and Yang, 2010 **syn. nov.** = *B.hildebrandtae* Pleske, 1930. The authors also provide an identification key, descriptions, photographs and distributional notes on these Korean species.

## Introduction

The genus *Beris* Latreille is a representative group of soldier flies belonging to the subfamily Beridinae within the family Stratiomyidae (Rozkošný 1998). Latreille (1802) erected the genus, based on the type species *Stratiomyssexdentata* Fabricius, 1781 [= *Berischalybata* (Forster, 1771)] from England (Forster 1771, Rozkošný and Nartshuk 1988). To date, 49 described members of the genus *Beris* Latreille have been recorded throughout the world (Woodley 2001, Woodley 2011, Yang et al. 2014). Most recently, *Berisnigra* Meigen, 1820 was resurrected to species rank and is now treated as the senior synonym of *B.hauseri* Stuke, 2004 by Zeegers (2021). The larvae of *Beris* are saproxylic and terrestrial. They usually spend this life stage under decaying organic matter, wet moss, compost and wood debris (Woodley 1995). The adults are normally found in foliage near mountain valleys, marshes and other damp places (Rozkošný 1973).

Prior to this study, altogether only three species under two genera of the subfamily Beridinae had been recorded in the Korean fauna, viz. *Actinadiadema* Lindner, 1936, *Actinajezoensis* (Matsumura, 1916) and *Allognostavagans* (Loew, 1873) (National Institute of Biological Resources 2019). As a result of field surveys from Gangwon-do and Gyeongsangbuk-do Provinces in Korea, the authors discovered three unknown species and identified that they belong to *Beris*, in addition to an unrecorded genus from Korea. Based on the present results, the current knowledge of Korean Beridinae includes six species within three genera. The morphological features for discriminating these taxa, as well as images of the genitalia of each species, are presented below. A key for the separation of Korean *Beris* and nomenclatural changes for the two East Asian species are also provided.

## Materials and methods

The voucher specimens of *Beris* provided in this study were collected using both sweeping and Malaise traps. Collected specimens were deposited in the Laboratory of Systematic Entomology at Kyungpook National University, Daegu, Korea. The terminology used for describing the morphological external features and genital structures generally follows Cumming and Wood (2017) and Yang et al. (2014), respectively. The descriptions provided herein were mainly based on Korean specimens. Since external variations have been reported in some species depending on the distributional range, the authors only discussed the morphological variation of materials from the Korean Peninsula.

To investigate the male genital complex, the dissected distal part of the abdomen was macerated in 10% potassium hydroxide (KOH) solution and transferred to glycerine jelly for visualisation and photographs. The genital structures were then visualised using either a stereomicroscope (Olympus SZX16) or a compound microscope (Olympus BX50). A series of images were acquired using an Olympus digital camera (DP 71) and these were then compiled into a single in-depth figure using Helicon Focus 7.0.2 software (Son and Suh 2020, Lee and Suh 2020).

### Acronyms for depositories

[CAU] Entomological Museum, China Agricultural University, China.

[KU] Entomological Laboratory, Kyushu University, Japan.

[MNHN] Entomology Laboratory, National Museum of Natural History, France.

[SCI] Laboratory of Taxonomy and Ecology, Institute of Entomology, Academia Sinica, China.

[UZMH] Zoological Museum, Finnish Museum of Natural History, University of Helsinki, Finland.

[ZMAS] Zoological Institute, Academy of Sciences, Russia.

## Taxon treatments

### Beris

Latreille, 1802

B7ABF4F4-E944-5386-B38A-7EB58D770F6D

Beris Latreille, 1802 - *Latreille 1802*: 447 (Type-species: *Stratiomyssexdentata* Fabricius).
Hexacantha Meigen, 1803 - Meigen 1803: 264 (Type-species: *Muscaclavipes* Linnaeus).
Octacantha Lioy, 1864 - Lioy 1864: 586 (Type-species: *Berisfuscipes* Meigen).
Hemiberis Enderlein, 1921 - Enderlein 1921: 209 (Type-species: *Berisquadridentata* Walker).

#### Diagnosis

Unlike other genera in this subfamily, this genus has a degenerated maxillary palpus and the scape and pedicel have almost the same length. In males, hind tarsomere 1 is particularly swollen (about two times wider than the hind tibia).

See following references for details: Nagatomi and Tanaka (1972), Woodley (1981), Rozkošný (1982), Yang and Nagatomi (1992), Woodley (1995), Yang et al. (2014).

### Beris fuscipes

Meigen, 1820

D5003E28-431D-59C8-838E-546116D4E49B

Beris fuscipes Meigen, 1820 - Meigen 1820: 8 (Type-locality: England). ♂ Holotype [MNHN].
Beris sachalinensis Pleske, 1926 - Pleske 1926: 408 (Type-locality: Russia). ♂ Lectotype [ZMAS], synonymised by Pleske 1930.
Beris fuscotibialis Pleske, 1926 - Pleske 1926: 409 (Type-locality: Russia). ♂ Lectotype [ZMAS], synonymised by Nartshuk and Rozkošný 1976.
Beris sychuanensis Pleske, 1926 - Pleske 1926: 411 (Type-locality: China). ♂ Lectotype [ZMAS], synonymised by Nartshuk and Rozkošný 1976.
Beris petiolata Frey, 1961 - Frey 1961: 80 (Type-locality: Japan). 4♀ Syntypes [UZMH], synonymised by Nagatomi and Tanaka 1972.

#### Materials

- **Type status:** Other material. **Occurrence:** individualCount: 1; sex: 1 male; lifeStage: adult; **Taxon:** scientificName: *Berisfuscipes*; **Location:** country: Republic of Korea; stateProvince: Gangwon-do; locality: Pyeongchang-gun, Jinbu-myeon, Jangjeon-ri, Mt. Gariwangsan, 37°28'29"N, 128°31'59"E; **Identification:** identifiedBy: J Lee; dateIdentified: 2021; **Event:** samplingProtocol: malaise trap; eventDate: V/30/2020; **Record Level:** language: en; institutionCode: KNU; basisOfRecord: PreservedSpecimen
- **Type status:** Other material. **Occurrence:** individualCount: 4; sex: 4 males; lifeStage: adult; **Taxon:** scientificName: *Berisfuscipes*; **Location:** country: Republic of Korea; stateProvince: Gangwon-do; locality: Pyeongchang-gun, Daegwallyeong-myeon, Hoenggye-ri, Seonjaryeong, 37°41'45"N, 128°45'15"E; **Identification:** identifiedBy: J Lee; dateIdentified: 2021; **Event:** samplingProtocol: malaise trap; eventDate: VI/04/2021; **Record Level:** language: en; institutionCode: KNU; basisOfRecord: PreservedSpecimen

#### Description

**Figure 1.**
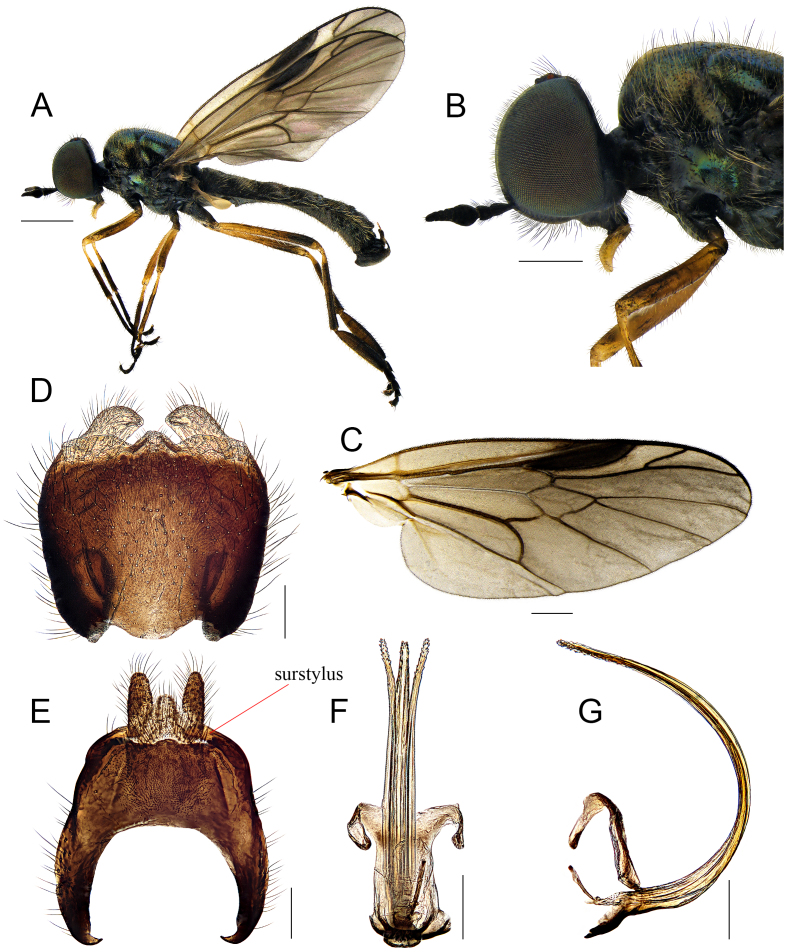
*Berisfuscipes* Meigen, 1820: **A.** Male habitus, lateral view; **B.** Male head, lateral view; **C.** Male wing; **D.** Genital capsule, dorsal view; **E.** Epandrium, cerci and proctiger, dorsal view; **F.** Phallus, dorsal view; **G.** Ditto, lateral view. Scale bars: A = 1.0 mm; B, C = 0.5 mm; D–G = 0.1 mm.

**Male.** Body length (excluding antennae): 6.5–7.4 mm; wing length: 5.5–6.1 mm. **Head**: Black and somewhat sub-shining; compound eyes densely covered with pale brown hairs; occiput and vertex with pale brown recumbent hairs; postocular areas towards lower margin of eye slightly pale pollinose; face black pilose; lower frons with short dark brown to black hairs; antennae short, extreme apex of pedicel slightly tinged with yellowish-brown; flagellum 1.3-1.4 times as long as scape and pedicel combined; flagellomeres 1-2 abruptly swollen, about 1.6-1.8 times wider than pedicel (Fig. 1B). **Thorax**: Metallic green, scutum tomentose with pale brown hairs; postpronotal lobe tinged with brown; anepisternum, except anterior and posterior part, lower part of katepisternum and anepimeron, nearly bare. **Legs**: Mainly yellowish-brown, but the following parts tinged dark brown to black: all coxae, extreme apex of mid-femur, apical half of hind femur, except extreme apex, mid-tibia, except base, middle portion of hind tibia and tarsi (base of fore tarsus slightly paler); hind tarsomere 1 about 1.8-1.9 times wider than hind tibia (Fig. 1A). **Wings**: Tinged with dark brown; base of M_1_ and M_2_ convergent (Fig. 1C). **Abdomen**: Dark brown to black, lateral margin of tergites with conspicuous long yellowish-brown to brown hairs; sterna partly with recumbent pale yellow to yellowish-brown hairs densely; epandrium broad, semi-circular-shaped, posterior margin practically straight; surstyli inserted posterolaterally, feebly curved inwards; cerci parallel-sided and straight; proctiger nearly triangular, apex somewhat rounded (Fig. 1E); gonocoxite wider than long, posteromedian projection not well-developed, only slightly protruding; gonostyli blunt, not tapering (Fig. 1D); phallus slender, tripartite; lateral lobes slightly divergent from and slightly longer than median lobe (Fig. 1F and G).

Female: Specimen unavailable.

#### Diagnosis

This species can be distinguished from other congeners by the following key diagnostic characters: flagellum short, basal flagellomeres 1-2 (or 1-3) abruptly swollen in both sexes (Fig. 1B), posterior margin of epandrium with well-developed surstyli (males) (Fig. 1E); frons about 1/5 to 1/4 of head-width (females) in anterior view (Rozkošný and Nartshuk 1980:416). This species is particularly similar to the nominate Palearctic species, *B.chalybata* (Forster, 1771) and *B.strobli* Dusek & Rozkosny, 1968, but the flagellum of the latter two species is long and not thickened basally, male genitalia without surstyli and the width of frons in the female is about 1/3 of head-width (Rozkošný 1982).

#### Distribution

Korea (new record: Gangwon-do), China (Sichuan, Ningxia, Gansu), Japan (Hokkaido, Honshu), Russia (Far East: Siberia), Mongolia, Europe, Canada and USA.

#### Taxon discussion

According to literature, this species has been reported to have intraspecific variation in the ground colour of antennal segments, hairs on head and thorax, parts of the legs and in wing venation and shape of surstylus (Woodley 1981, Rozkošný 1982, Woodley 1995). In the Korean specimens, these features are as follows: antennae black, but extreme apex of pedicel yellowish-brown; hairs on frons, face and scutum mainly pale brown to black; M_1_ and M_2_ touching proximally, M_3_ invisible; basal part of femora and tibiae tinged with yellow; surstyli not straight, but curved inwards.

#### Notes

This Holarctic species is widely distributed in large regions of the Palearctic, Europe to Asia and Nearctic realms, North America. The distributional ranges are as follows: Palearctic: Europe [Central: Austria, Czech Republic, Germany, Hungary, Poland, Slovakia, Switzerland; Northern: Finland, Norway, Sweden; Southern: Italy; Western: France, UK, Ireland; Eastern: Georgia, Roumania, Ukraine] and Asia [Central: Kazakhstan; East: China, Japan, Mongolia, Russia (Far East)]; Nearctic: Canada and USA (Rozkošný 1982, Woodley 2001).

### Beris hildebrandtae

Pleske, 1930

278E33C0-8A08-52AB-A298-0AC25DF7C5C3

Beris hildebrandtae Pleske, 1930 - Pleske 1930: 195 (Type-locality: Russia). ♂ Lectotype [ZMAS].
Beris angustifacies Nagatomi and Tanaka, 1972 - Nagatomi and Tanaka 1972: 91 (Type-locality: Japan). ♂ Holotype [KU]. **New synonymy.**
Beris liaoningana Cui et al, 2010 - Cui et al. 2010: 280 (Type-locality: China). ♂ Holotype [CAU]. **New synonymy.**

#### Materials

- **Type status:** Other material. **Occurrence:** individualCount: 1; sex: 1 male; lifeStage: adult; **Taxon:** scientificName: Berishildebrandtae; **Location:** country: Republic of Korea; stateProvince: Gangwon-do; locality: Samcheok-si, Hajang-myeon, Galjeon-ri, Mt. Jungbongsan, 37°24'04"N, 128°53'51"E; **Identification:** identifiedBy: J Lee; dateIdentified: 2021; **Event:** samplingProtocol: sweeping; eventDate: VII/26/2018; **Record Level:** language: en; institutionCode: KNU; basisOfRecord: PreservedSpecimen
- **Type status:** Other material. **Occurrence:** individualCount: 6; sex: 1 male, 5 females; lifeStage: adult; **Taxon:** scientificName: Berishildebrandtae; **Location:** country: Republic of Korea; stateProvince: Gangwon-do; locality: Jeongseon-gun, Yeoryang-myeon, Gujeol-ri, Mt. Sangwonsan, 37°32'18"N, 128°39'1"E; **Identification:** identifiedBy: J Lee; dateIdentified: 2021; **Event:** samplingProtocol: sweeping; eventDate: VIII/10/2021; **Record Level:** language: en; institutionCode: KNU; basisOfRecord: PreservedSpecimen
- **Type status:** Other material. **Occurrence:** individualCount: 5; sex: 5 females; lifeStage: adult; **Taxon:** scientificName: Berishildebrandtae; **Location:** country: Republic of Korea; stateProvince: Gangwon-do; locality: Pyeongchang-gun, Jinbu-myeon, Jangjeon-ri, Mt. Gariwangsan, 37°28'29"N, 128°31'59"E; **Identification:** identifiedBy: J Lee; dateIdentified: 2021; **Event:** samplingProtocol: sweeping; eventDate: VIII/12/2021; **Record Level:** language: en; institutionCode: KNU; basisOfRecord: PreservedSpecimen

#### Description

**Figure 2.**
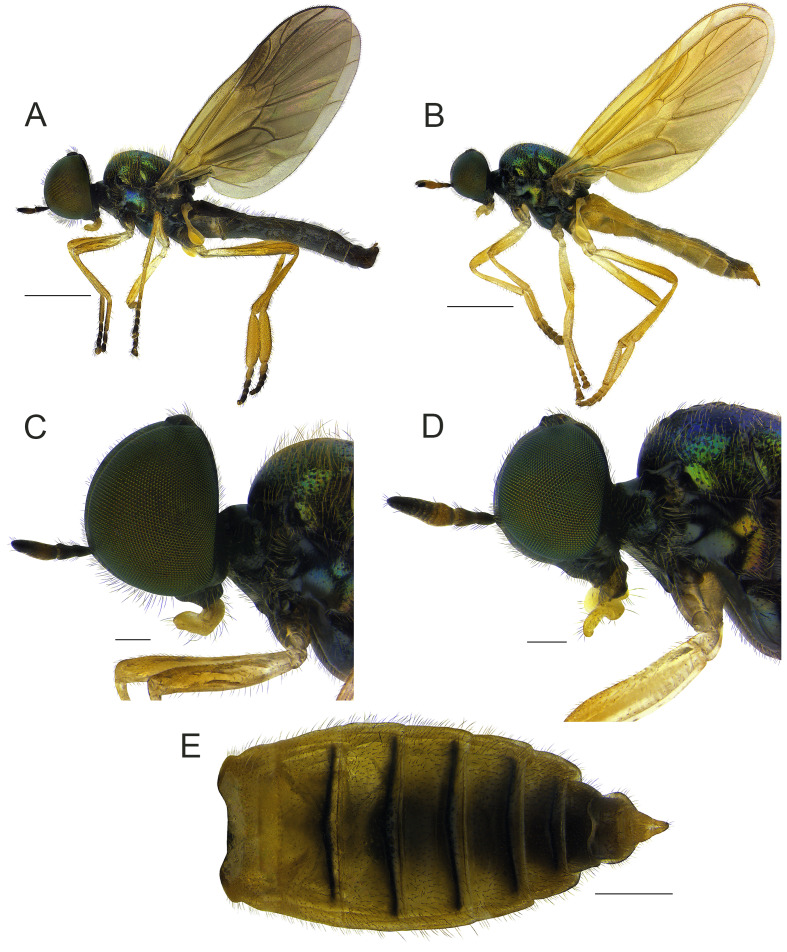
*Berishildebrandtae* Pleske, 1930: **A** Male habitus, lateral view; **B** Female habitus, lateral view; **C** Male head, lateral view; **D** Female head, lateral view; **E** Female abdomen, dorsal view. Scale bars: A, B = 1.0 mm; C, D = 0.2 mm; E = 0.5 mm.

**Figure 3.**
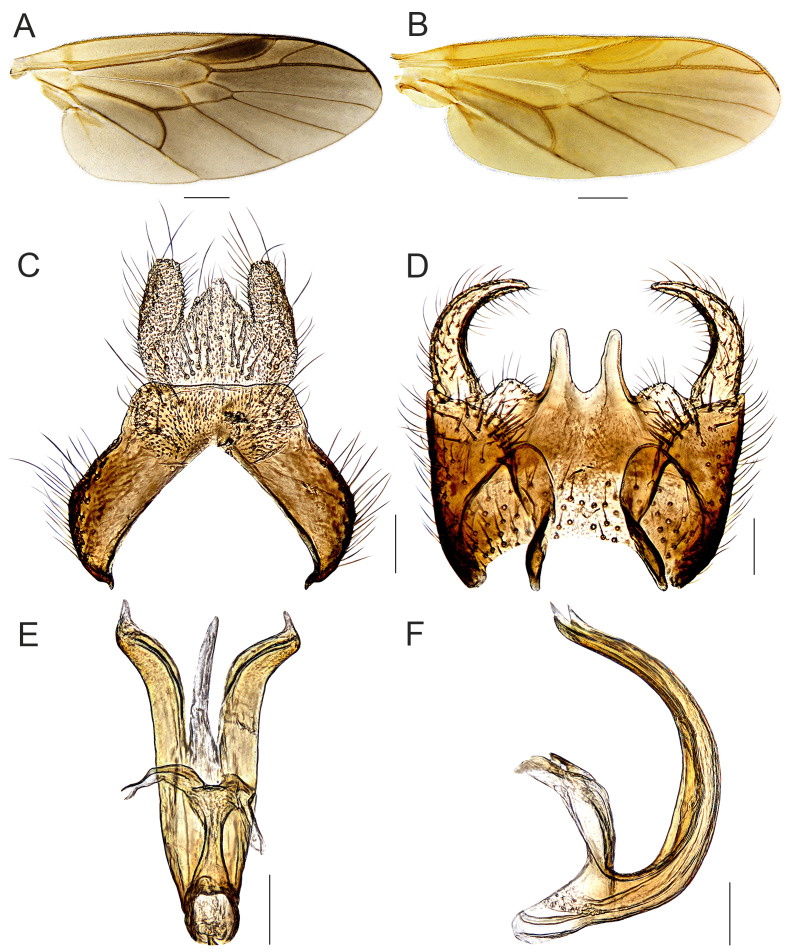
*Berishildebrandtae* Pleske, 1930: **A** Male wing; **B** Female wing; **C** Epandrium, cerci and proctiger, dorsal view; **D** Genital capsule, dorsal view; **E** Phallus, dorsal view; **F** Ditto, lateral view. Scale bars: A, B = 0.5 mm; C–F = 0.1 mm.

**Male.** Body length (excluding antennae): 5.3-5.4 mm; wing length: 5.0-5.1 mm. **Head**: Black and shining; compound eyes densely covered with black hairs; occiput and vertex with pale recumbent hairs; postocular areas towards lower margin of eye covered with pale yellow hairs; face mixed yellow and black hairs; lower frons with short pale hairs; antennae short, extreme apex of pedicel tinged yellowish-brown, both surfaces of flagellomeres 2-4 and inner surface of flagellomeres 5-6 tinged with yellowish-brown to reddish-brown; flagellum 1.4-1.5 times as long as scape and pedicel combined; basal flagellomeres not abruptly swollen (Fig. 2C). **Thorax**: Metallic green, scutum tomentose with yellow hairs; postpronotal lobe tinged with yellowish-brown; central part of anepisternum, katepisternum, except upper part and posterior part of anepimeron, nearly bare. **Legs**: Mainly yellow, but the following parts tinged dark brown: all coxae, except at extreme apex, fore and mid-tarsomeres 2-5, hind tarsomeres 3-5; all trochanters and basal half of femora pale; hind tarsomere 1 about 1.8-2.0 times wider than hind tibia (Fig. 2A). **Wings**: Tinged with brown; base of M_1_ and M_2_ separated (Fig. 3A). **Abdomen**: Dark brown, lateral margin of tergites mixed with conspicuous long pale yellow and black hairs; sterna wholly densely covered with recumbent yellowish-brown hairs; epandrium broad, anterior margin triangular, posterior margin straight; surstyli absent; cercus parallel-sided and straight; proctiger equilaterally triangular (Fig. 3C); gonocoxite wider than long, median projection on gonocoxite well-developed, bilobate with deep incision; gonostyli clearly curved inwards, tapering apically (Fig. 3D); phallus comparatively stout, tripartite; lateral lobes fairly divergent and with a short straight and pointed apex, somewhat longer than median lobe; median lobe broader at base, more slender and curved distally (Fig. 3E and F).

**Female.** Similar to males, but differing as follows: Body length (excluding antennae): 5.0–5.8 mm; wing length: 4.2–5.0 mm. **Head**: Hairs on compound eyes relatively shorter and fewer than in males; postocular area with distinct pale pruinosity along towards lower margin of eye; antennae mostly tinged reddish-brown, flagellomeres 6-8 dark brown to black; flagellum 1.7-2.0 times as long as scape and pedicel combined; terminal flagellomere more pointed apically (Fig. 2D). **Thorax**: Hairs on side of scutum shorter than in males. **Legs**: All coxae completely pale to pale yellow; hind tarsomere 1 less swollen than in males (Fig. 2B). **Wings**: Tinged with pale yellow (Fig. 3B). **Abdomen**: Yellow to yellowish-brown and generally glossy; hairs on lateral margin of tergites shorter than in males; posterior margins of tergites 2-6 with transverse black stripes; middle of tergites 2-6 tinged with brown; sterna normally pale yellow, but middle part of sternites 2-5 sometimes dark brown; sternites wholly densely covered with recumbent pale yellow hairs (Fig. 2E).

#### Diagnosis

This species can be distinguished from other congeners by the following key diagnostic characters: legs, except coxae and tarsomeres 2-5, mainly pale yellow to yellow (males) (Fig. 2A); wings tinged with pale yellow (Fig. 3B), posterior margin of tergites 2-6 with transverse black stripes (females) (Fig. 2E) (Nartshuk and Rozkošný 1975).

#### Distribution

Korea (new record: Gangwon-do), China (Liaoning), Japan (Hokkaido, Honshu) and Russia (Far East: Siberia).

#### Taxon discussion

This species was discovered by Pleske in 1930 from the Russian Far East and 45 years later, it was re-described in detail by Russian entomologists, Nartshuk and Rozkošný (1975). It has noticeable intraspecific variations, including sexual dimorphism (see Figs 2, 3). Although there are some variations, i.e. colouration of antennal segments 2-3 and coxae, the Korean specimens collected in Gangwon-do Province highly matched with the original description and re-description (Pleske 1930; Nartshuk and Rozkošný 1975) and we identified these materials as *B.hildebrandtae* Pleske. In the case of *B.liaoningana* Cui, Li and Yang, 2010, this species was described, based on the difference of the phallus structures, i.e. the gap, relative length and divergences of three lobes, in comparison with *B.hildebrandtae* Pleske. However, in the process of genital examination for Korean materials, we confirmed that the phallus structures of *B.hildebrandtae* Pleske appeared very similar to the drawing made by Cui et al. (2010) depending on the observation angle in glycerine jelly. When drawing a three-dimensional genital structure like a phallus complex, it often causes some distortions, which leads to the taxonomic problem that the same species are described as different species. Furthermore, the external morphology (except variations) and the other genital characters of this Chinese species significantly agreed with the re-description of *B.hildebrandtae* Pleske. Therefore, there is no doubt that *B.liaoningana* Cui, Li et Yang is identical with *B.hildebrandtae* Pleske. As for *B.angustifacies* Nagatomi and Tanaka, 1972, it was described, based on specimens from Japan and Russian Far East without comparison with *B.hildebrandtae* Pleske (Nagatomi and Tanaka 1972). They also illustrated *B.angustifacies* as having a bifurcate phallus, which is different from *B.hildebrandtae* (tripartite phallus). However, Nartshuk and Rozkošný (1975) included the personal communication of Nagatomi that “*B.angustifacies* Nagatomi and Tanaka is probably separated from *B.hildebrandtae* Pleske by the female’s abdominal colour (*B.angustifacies* more darkened) and these two species are very similar or possibly conspecific”. The authors confirmed that the abdomens of several Korean female specimens were also partially yellowish-brown to brown. Moreover, as in the example of *Berisclavipes* (Linnaeus, 1767), there is a possible intraspecific variation in the colour of the abdomen (Rozkošný 1982). In other words, the colour of the abdomen is not the main character to distinguish species-level. In conclusion, it is reasonable to treat *B.angustifacies* as a junior synonym of *B.hildebrandtae* Pleske.

#### Notes

The males of this species show intraspecific variations on their thoracic hairs, colour of coxae and tarsi. In the Korean specimens, these features are as follows: thoracic hairs usually pale yellow to yellow; all coxae dark brown to black, but extreme apices yellow, mid-coxa sometimes completely pale; tarsi mainly yellow, but fore and mid-tarsomeres 2-5 and hind tarsomeres 3-5 dark brown. The females of this species also have some variations on their colouration of antennae, darkening on abdomen and wing venation. For Korean materials, the antennae are mostly tinged with yellow to reddish-brown, but flagellomeres 6-8 dark brown to black. Additionally, the following variations are occasionally noted in some individuals from Korea: scape and pedicel tinged with reddish-brown and yellowish-brown, respectively; extreme base of scape dark brown to black; flagellomere 6 orange brown, not darkened like flagellomeres 7-8. The abdomens of Korean materials are mainly yellow to yellowish-brown and middle of tergites 2-7 more darkened; these segments are fully dark brown or pale yellow depending on individuals. Furthermore, M_1_ and M_2_ are generally well-separated proximally, but in some individuals, these veins touch at their bases.

### Beris hirotui

Ôuchi, 1943

E775A3E7-AAD5-5619-8503-C86B2CA7BAD9

Beris hirotui Ôuchi, 1943 - Ôuchi 1943: 487 (Type-locality: Japan). ♀ Holotype [SCI].
Beris hisotui (sic): Ôuchi 1943: 487.
Beris hirotsui (sic): Nagatomi and Tanaka 1972: 98; Nartshuk and Rozkošný 1975: 85; Rozkošný and Nartshuk 1988: 46; Yang and Nagatomi 1992: 167; Li et al. 2009: 130.

#### Materials

- **Type status:** Other material. **Occurrence:** individualCount: 3; sex: 3 males; lifeStage: adult; **Taxon:** scientificName: *Berishirotui*; **Location:** country: Republic of Korea; stateProvince: Gyeongsangbuk-do; locality: Gunwi-gun, Bugye-myeon, Dongsan-ri, Mt. Palgongsan, 36°01'46"N, 128°40'28"E; **Identification:** identifiedBy: J Lee; dateIdentified: 2020; **Event:** samplingProtocol: sweeping; eventDate: V/27/2020; **Record Level:** language: en; institutionCode: KNU; basisOfRecord: PreservedSpecimen
- **Type status:** Other material. **Occurrence:** individualCount: 1; sex: 1 male; lifeStage: adult; **Taxon:** scientificName: *Berishirotui*; **Location:** country: Republic of Korea; stateProvince: Gangwon-do; locality: Yeongwol-gun, Yeongwol-eup, Samok-ri, Donggang River, 37°13'57"N, 128°30'55"E; **Identification:** identifiedBy: J Lee; dateIdentified: 2020; **Event:** samplingProtocol: malaise trap; eventDate: V/29/2020; **Record Level:** language: en; institutionCode: KNU; basisOfRecord: PreservedSpecimen
- **Type status:** Other material. **Occurrence:** individualCount: 4; sex: 3 males, 1 female; lifeStage: adult; **Taxon:** scientificName: *Berishirotui*; **Location:** country: Republic of Korea; stateProvince: Gangwon-do; locality: Jeongseon-gun, Yeoryang-myeon, Gujeol-ri, Mt. Sangwonsan, 37°32'18"N, 128°39'15"E; **Identification:** identifiedBy: J Lee; dateIdentified: 2020; **Event:** samplingProtocol: sweeping; eventDate: V/30/2020; **Record Level:** language: en; institutionCode: KNU; basisOfRecord: PreservedSpecimen
- **Type status:** Other material. **Occurrence:** individualCount: 1; sex: 1 male; lifeStage: adult; **Taxon:** scientificName: *Berishirotui*; **Location:** country: Republic of Korea; stateProvince: Gangwon-do; locality: Pyeongchang-gun, Jinbu-myeon, Jangjeon-ri, Mt. Gariwangsan, 37°27'59"N, 128°32'18"E; **Identification:** identifiedBy: J Lee; dateIdentified: 2020; **Event:** samplingProtocol: malaise trap; eventDate: V/30/2020; **Record Level:** language: en; institutionCode: KNU; basisOfRecord: PreservedSpecimen
- **Type status:** Other material. **Occurrence:** individualCount: 31; sex: 8 males, 23 females; lifeStage: adult; **Taxon:** scientificName: *Berishirotui*; **Location:** country: Republic of Korea; stateProvince: Gangwon-do; locality: Pyeongchang-gun, Daegwallyeong-myeon, Hoenggye-ri, Seonjaryeong, 37°41'45"N, 128°45'15"E; **Identification:** identifiedBy: J Lee; dateIdentified: 2020; **Event:** samplingProtocol: sweeping; eventDate: V/31/2020; **Record Level:** language: en; institutionCode: KNU; basisOfRecord: PreservedSpecimen
- **Type status:** Other material. **Occurrence:** individualCount: 1; sex: 1 male; lifeStage: adult; **Taxon:** scientificName: *Berishirotui*; **Location:** country: Republic of Korea; stateProvince: Gangwon-do; locality: Jeongseon-gun, Yeoryang-myeon, Gujeol-ri, Mt. Nochusan, 37°31'08"N, 128°46'42"E; **Identification:** identifiedBy: J Lee; dateIdentified: 2021; **Event:** samplingProtocol: sweeping; eventDate: V/20/2021; **Record Level:** language: en; institutionCode: KNU; basisOfRecord: PreservedSpecimen
- **Type status:** Other material. **Occurrence:** individualCount: 2; sex: 2 males; lifeStage: adult; **Taxon:** scientificName: *Berishirotui*; **Location:** country: Republic of Korea; stateProvince: Gangwon-do; locality: Jeongseon-gun, Yeoryang-myeon, Yeoryang-ri, Mt. Banryusan, 37°27'05"N, 128°44'08"E; **Identification:** identifiedBy: J Lee; dateIdentified: 2021; **Event:** samplingProtocol: sweeping; eventDate: V/21/2021; **Record Level:** language: en; institutionCode: KNU; basisOfRecord: PreservedSpecimen
- **Type status:** Other material. **Occurrence:** individualCount: 1; sex: 1 female; lifeStage: adult; **Taxon:** scientificName: *Berishirotui*; **Location:** country: Republic of Korea; stateProvince: Gangwon-do; locality: Pyeongchang-gun, Jinbu-myeon, Jangjeon-ri, Mt. Gariwangsan, 37°27'59"N, 128°32'18"E; **Identification:** identifiedBy: J Lee; dateIdentified: 2021; **Event:** samplingProtocol: sweeping; eventDate: V/22/2021; **Record Level:** language: en; institutionCode: KNU; basisOfRecord: PreservedSpecimen
- **Type status:** Other material. **Occurrence:** individualCount: 14; sex: 10 males, 4 females; lifeStage: adult; **Taxon:** scientificName: *Berishirotui*; **Location:** country: Republic of Korea; stateProvince: Daegu-si; locality: Gachang-eup, Jeongdae-ri, Mt. Biseulsan, 35°43'54"N, 128°32'53"E; **Identification:** identifiedBy: J Lee; dateIdentified: 2021; **Event:** samplingProtocol: sweeping; eventDate: V/30/2021; **Record Level:** language: en; institutionCode: KNU; basisOfRecord: PreservedSpecimen
- **Type status:** Other material. **Occurrence:** individualCount: 3; sex: 2 males, 1 female; lifeStage: adult; **Taxon:** scientificName: *Berishirotui*; **Location:** country: Republic of Korea; stateProvince: Gangwon-do; locality: Pyeongchang-gun, Jinbu-myeon, Duil-ri, Mt. Odaesan, 37°41'09"N, 128°34'25"E; **Identification:** identifiedBy: J Lee; dateIdentified: 2021; **Event:** samplingProtocol: sweeping; eventDate: VI/04/2021; **Record Level:** language: en; institutionCode: KNU; basisOfRecord: PreservedSpecimen
- **Type status:** Other material. **Occurrence:** individualCount: 22; sex: 7 males, 15 females; lifeStage: adult; **Taxon:** scientificName: *Berishirotui*; **Location:** country: Republic of Korea; stateProvince: Gangwon-do; locality: Pyeongchang-gun, Daegwallyeong-myeon, Hoenggye-ri, Seonjaryeong, 37°41'45"N, 128°45'15"E; **Identification:** identifiedBy: J Lee; dateIdentified: 2021; **Event:** samplingProtocol: sweeping; eventDate: VI/04/2021; **Record Level:** language: en; institutionCode: KNU; basisOfRecord: PreservedSpecimen
- **Type status:** Other material. **Occurrence:** individualCount: 2; sex: 2 males; lifeStage: adult; **Taxon:** scientificName: *Berishirotui*; **Location:** country: Republic of Korea; stateProvince: Gangwon-do; locality: Pyeongchang-gun, Daegwallyeong-myeon, Yongsan-ri, Mt. Barwangsan, 37°38'18"N, 128°40'09"E; **Identification:** identifiedBy: J Lee; dateIdentified: 2021; **Event:** samplingProtocol: sweeping; eventDate: VI/05/2021; **Record Level:** language: en; institutionCode: KNU; basisOfRecord: PreservedSpecimen
- **Type status:** Other material. **Occurrence:** individualCount: 5; sex: 4 males, 1 female; lifeStage: adult; **Taxon:** scientificName: *Berishirotui*; **Location:** country: Republic of Korea; stateProvince: Gangwon-do; locality: Jeongseon-gun, Hwaam-myeon, Morun-ri, Mt. Gwangdaesan, 37°18'52"N, 128°49'10"E; **Identification:** identifiedBy: J Lee; dateIdentified: 2021; **Event:** samplingProtocol: sweeping; eventDate: VI/09/2021; **Record Level:** language: en; institutionCode: KNU; basisOfRecord: PreservedSpecimen
- **Type status:** Other material. **Occurrence:** individualCount: 3; sex: 3 males; lifeStage: adult; **Taxon:** scientificName: *Berishirotui*; **Location:** country: Republic of Korea; stateProvince: Gangwon-do; locality: Jeongseon-gun, Nam-myeon, Mureung-ri, Mt. Duwibong, 37°14'05"N, 128°45'35"E; **Identification:** identifiedBy: J Lee; dateIdentified: 2021; **Event:** samplingProtocol: sweeping; eventDate: VI/10/2021; **Record Level:** language: en; institutionCode: KNU; basisOfRecord: PreservedSpecimen
- **Type status:** Other material. **Occurrence:** individualCount: 6; sex: 5 males, 1 female; lifeStage: adult; **Taxon:** scientificName: *Berishirotui*; **Location:** country: Republic of Korea; stateProvince: Gangwon-do; locality: Jeongseon-gun, Sabuk-eup, Sabuk-ri, Mt. Baekunsan, 37°11'23"N, 128°48'38"E; **Identification:** identifiedBy: J Lee; dateIdentified: 2021; **Event:** samplingProtocol: sweeping; eventDate: VI/10/2021; **Record Level:** language: en; institutionCode: KNU; basisOfRecord: PreservedSpecimen

#### Description

**Figure 4.**
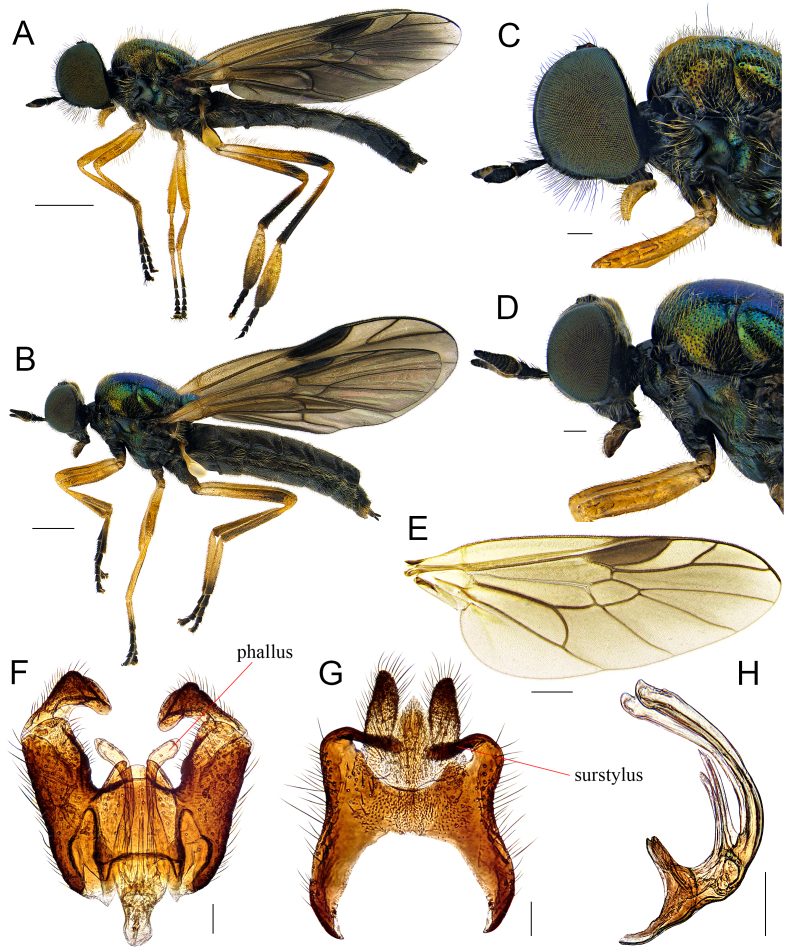
*Berishirotui* Ôuchi, 1943: **A** Male habitus, lateral view; **B** Female habitus, lateral view; **C** Male head, lateral view; **D** Female head, lateral view; **E** Male wing; **F** Genital capsule, dorsal view; **G** Epandrium, cerci and proctiger, dorsal view; **H** Phallus, lateral view. Scale bars: A, B = 1.0 mm; C, D = 0.2 mm; E = 0.5 mm; F–H = 0.1 mm.

**Male.** Body length (excluding antennae): 5.9–7.4 mm; wing length: 5.0–6.0 mm. **Head**: Black and subshining; compound eyes densely covered with pale yellow hairs; occiput and vertex with pale recumbent hairs; postocular areas towards lower margin of eye slightly pale pollinose; face black pilose; lower frons with short black hairs; antennae short, apex of pedicel and inner surface of flagellomeres 2-6 tinged with pale yellow; flagellum 1.1-1.2 times as long as scape and pedicel combined; flagellomeres 3-6 swollen, about 1.5-1.6 times wider than pedicel (Fig. 4C). **Thorax**: Metallic green, scutum tomentose with pale yellow hairs; postpronotal lobe tinged with yellow; central part of anepisternum, katepisternum, except upper part and meron nearly bare. **Legs**: Mostly yellowish-brown, but the following parts tinged dark brown: fore and hind coxa, except apex, apical portion of hind femur, except extreme apex, hind tibia, except base, apices of tarsomeres 1 (but hind one slightly pale), and tarsomeres 2-5; hind tarsomere 1 about 2.4-2.6 times wider than hind tibia (Fig. 4A). **Wings**: Tinged with yellowish-brown; base of M_1_ and M_2_ convergent (Fig. 4E). **Abdomen**: Dark brown to black, lateral margin of tergites with conspicuously long pale yellow hairs; sterna wholly densely covered with recumbent pale yellow hairs; epandrium long, median margin quite concave, anterior margin semicircular; long surstyli inserted posterolaterally, strongly curved inwards; cerci nearly parallel-sided and straight; proctiger narrow and triangular (Fig. 4G); gonocoxite longer than wide, median projection incised rather than protruding, subquadrate; gonostyli clearly bent inwards; phallus comparatively thick, bifurcate; lobes bent and with diverging apices, three dorsal processes present (Fig. 4F, H).

**Female.** Similar to males, but differing as follows: Body length (excluding antennae): 5.0–6.2 mm; wing length: 4.4–5.8 mm. **Head**: Hairs on compound eyes relatively shorter and fewer than in males; occiput properly visible in anterior view; inner surface of flagellum, except apex tinged with brown to reddish-brown; flagellum nearly 1.3-1.5 times as long as scape and pedicel combined (Fig. 4D). **Thorax**: Hairs on side of scutum shorter than in males. **Legs**: Distal part of hind tibia paler rather than dark brown; hind tarsomere 1 less swollen than in males (Fig. 4B). **Abdomen**: Hairs on lateral margin of tergites shorter than in males.

#### Diagnosis

This species can be distinguished from other congeners by the following key diagnostic characters: flagellum about 1.5 to 2 times as long as scape (Fig. 4C), hind tarsomere 1 about 2.5 times wider than hind tibia (Fig. 4A) (males); flagellomere 8 less than 2 times as long as wide (Fig. 4D) (females). This species is particularly similar to the Japanese species, *B.nebulosa* Nagatomi and Tanaka, 1972, but the latter species has characteristics as follows: flagellum about 2.4 times as long as scape, hind tarsomere 1 as wide as hind tibia (males); flagellomere 8 more than 2 times as long as wide (Nagatomi and Tanaka 1972:112).

#### Distribution

Korea (new record: Gangwon-do, Gyeongsangbuk-do, Daegu-si), China (Hubei, Sichuan), Japan (Hokkaido, Honshu, Shikoku, Kyushu), Russia (Far East: Siberia) and Taiwan.

#### Notes

See Nagatomi and Tanaka 1972 for detailed description.

## Identification Keys

### Identification key to the Korean *Beris* species

**Table:** 

1	Compound eye holoptic; hind tarsomere 1 distinctly swollen (male)	2
–	Compound eye dichoptic; hind tarsomere 1 less swollen (female)	4
2	Hind femur and tibia completely pale yellow (unicoloured)	*B.hildebrandtae* Pleske
–	Hind femur and tibia mixed with yellow and black (bicoloured)	3
3	Basal segments of flagellum (especially flagellomeres 1-2) abruptly broadened	*B.fuscipes* Meigen
–	Basal segments of flagellum not abruptly broadened	*B.hirotui* Ôuchi
4	Ground colour of abdomen yellow to yellowish-brown; posterior margin of tergites 2-6 with transverse black stripes	*B.hildebrandtae* Pleske
–	Ground colour of abdomen wholly dark brown to black	5
5	Flagellum subequal with scape and pedicel combined; basal flagellomeres abruptly broadened	*B.fuscipes* Meigen
–	Flagellum about 1.3-1.5 times as long as scape and pedicel combined; basal flagellomeres not abruptly broadened	*B.hirotui* Ôuchi

The presented key is mainly based on external morphology of materials from specific localities (Gangwon-do and Gyeongsangbuk-do Provinces). For male genital characters of each species, see Figs 1, 3, 4.

## Discussion

**Figure 5.**
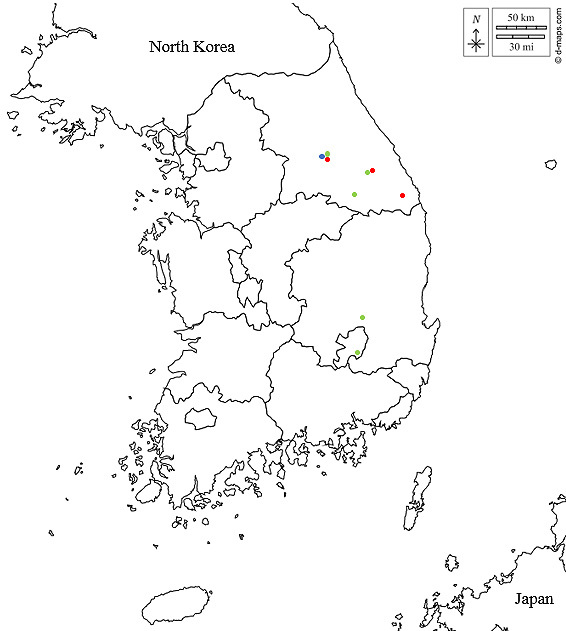
Distribution of *Beris* spp. from Korea. Blue circular spot: *Berisfuscipes* Meigen, 1820; Red circular spot: *Berishildebrandtae* Pleske, 1930; Green circular spot: *Berishirotui* Ôuchi, 1943. Base maps of South Korea: © 2007–2022 https://d-maps.com (accessed on 14 May 2022).

The results of our taxonomic study of the Korean Beridinae revealed the presence of three species previously unknown in the region, *Berisfuscipes* Meigen, 1820, *B.hildebrandtae* Pleske, 1930 and *B.hirotui* Ôuchi, 1943. Furthermore, this paper also proposes that two East Asian species, *B.angustifacies* Nagatomi and Tanaka, 1972 **syn. nov.** and *B.liaoningana* Cui, Li and Yang, 2010 **syn. nov.** be established as junior synonyms of *B.hildebrandtae* Pleske, 1930, thereby reducing the genus to a total of 47 valid species. The three species in this paper were mainly recorded from Gangwon-do Province, which borders North Korea. As such, these species are believed to inhabit North Korea as well (Fig. 5). Over the past decades, much research has been conducted on the species of *Beris* present in adjacent countries to Korea (i.e. China, Japan and Russia). The number of species and representative studies in each region are as follows: China [22 species: Yang and Nagatomi (1992), Li et al. (2009a), Li et al. (2009b), Cui et al. (2010), Qi et al. (2011), Li et al. (2011), Yang et al. (2014)]; Japan [6 species: Nagatomi and Tanaka (1972)]; Russia [13 species: Pleske (1926), Pleske (1930), Nartshuk and Rozkošný (1975), Nartshuk and Rozkošný (1976), Rozkošný and Nartshuk (1980)]. Accordingly, the Korean Peninsula is located in the centre of the above three countries, so it is considered that there are still many soldier flies awaiting discovery from Korea. Here, the authors present the *Beris* species likely to be found in the Korean Peninsula and their diagnostic features as well as distributional ranges.

*Beriscrassitarsis* Nagatomi and Tanaka, 1972

This species can be distinguished from other species of the *Beris* by the following characters: hind tarsomere 1 conspicuously swollen, about 3 times wider than hind tibia (males); last flagellomere 2.5-3.0 times as long as wide (females) (Nagatomi and Tanaka 1972). Distribution: Japan (Hokkaido and Honshu) and Russia (Far East: Siberia).

*Berislatifacies* Nagatomi and Tanaka, 1972

Only distinguished by male genital structures, as noted by Stuke (2004). Nagatomi and Tanaka (1972) described this new species from Japan and the Russian Far East. Some years later, Nartshuk and Rozkošný (1976) treated this species as a junior synonym of *Berisstrobli* Dusek & Rozkosny, 1968. However, this species was resurrected to species level by the study of Stuke (2004) (Woodley 2001, Woodley 2011, Martens et al. 2013). Distribution: Japan (Hokkaido and Honshu) and Russia (Far East: Siberia).

## Supplementary Material

XML Treatment for Beris

XML Treatment for Beris fuscipes

XML Treatment for Beris hildebrandtae

XML Treatment for Beris hirotui
